# Efficiency of Microwave Ablation for Cortisol-Producing Adrenal Adenomas: Case Series and a Literature Review

**DOI:** 10.15388/Amed.2025.32.2.5

**Published:** 2025-12-30

**Authors:** Alicija Krasavceva, Juozas Jarašūnas, Donatas Jocius, Romena Laukienė, Žydrūnė Visockienė, Virgilijus Beiša

**Affiliations:** 1 Faculty of Medicine, Vilnius University; 2 Department of Radiology, Nuclear Medicine and Medical Physics, Institute of Biomedical Sciences, Faculty of Medicine, Vilnius University; 3 Department of Radiology, Nuclear Medicine and Medical Physics, Institute of Biomedical Sciences, Faculty of Medicine, Vilnius University; 4 Center of Endocrinology, Vilnius University Hospital Santaros Klinikos; 5 Faculty of Medicine, Vilnius University; Center of Endocrinology, Vilnius University Hospital Santaros Klinikos; 6 Clinic of Gastroenterology, Nephrourology and Surgery, Faculty of Medicine, Vilnius University; Center of Abdominal Surgery, Vilnius University Hospital Santaros Klinikos

**Keywords:** adrenal adenoma, mild autonomous cortisol secretion, microwave ablation, Raktiniai žodžiai: antinksčių adenoma, lengva autonominė kortizolio sekrecija, mikrobangų abliacija

## Abstract

**Background:**

Adrenal adenomas, often discovered during imaging studies for unrelated conditions, pose diagnostic and therapeutic challenges due to their varying presentations and potential for hormone secretion. While surgical management remains the standard approach for hormonally active adrenal tumors, percutaneous ablation techniques – such as microwave ablation – have emerged as promising alternatives, particularly for patients who are not candidates for surgery.

**Materials and methods:**

This report presents a series of clinical cases in which percutaneous microwave ablation was used as an alternative to adrenalectomy. The study focuses on patients with cortisol-producing adrenal adenomas and explores the effectiveness of this minimally invasive procedure in achieving hormonal control and symptom relief.

**Results:**

Microwave ablation was successfully performed in all cases without major complications. Clinical follow-up demonstrated improvement in cortisol levels and relief of symptoms related to hypercortisolism. The procedure was well tolerated, offering an alternative therapeutic option for patients unsuitable for adrenalectomy.

**Conclusions:**

Percutaneous microwave ablation may serve as a safe and effective treatment option for cortisol-secreting adrenal adenomas in patients with Mild Autonomous Cortisol Secretion and ACTH-independent Cushing Syndrome who are not candidates for surgery. This approach may provide significant symptom relief and hormonal control with minimal invasiveness.

## Abstract (it)

**Įvadas:**

Antinksčių adenomų dažnai aptinkama atsitiktinai atliekant vaizdinius tyrimus dėl kitų nesusijusių priežasčių, o jų hormoninis aktyvumas ir įvairi klinikinė raiška gali kelti diagnostikos ir gydymo sunkumų. Nors chirurginis gydymas išlieka standartiniu funkciškai aktyvių antinksčių adenomų gydymo metodu, pastaruoju metu vis labiau populiarėja alternatyvūs ir minimaliai invazyvūs perkutaninės abliacijos metodai, tokie kaip mikrobangų abliacija – ypač pacientams, kurių chirurginis gydymas yra neįmanomas.

**Metodika:**

Šiame darbe aprašoma klinikinių atvejų, kai mikrobangų abliacija buvo taikyta kaip chirurginės adrenalektomijos alternatyva, serija siekiant įvertinti ir aptarti perkutaninės abliacijos efektyvumą gydant kortizolį sekretuojančias antinksčių adenomas.

**Rezultatai:**

Visiems pacientams mikrobangų abliacijos procedūros buvo techniškai sėkmingos, be didesnių komplikacijų. Buvo teigiamos ilgalaikės procedūrų baigtys – sumažėjo kortizolio ir gliukozės koncentracija kraujyje bei kiti su hiperkortizolizmu susiję klinikiniai požymiai. Mūsų atvejų serijos rezultatai atitinka publikuotus literatūros duomenis ir patvirtina, kad mikrobangų abliacija yra tinkama adrenalektomijos alternatyva gydant funkciškai aktyvias antinksčių adenomas.

**Išvados:**

Mikrobangų abliacija gali būti saugi ir efektyvi kortizolį sekretuojančių antinksčių adenomų gydymo alternatyva, ypač pacientams, sergantiems lengva autonomine kortizolio sekrecija ar AKTH nepriklausomu Kušingo sindromu, kai chirurginis gydymas yra kontraindikuotinas. Šis metodas gali užtikrinti reikšmingą simptomų palengvėjimą ir hormonų kontrolę, pasižymi mažesniu invazyvumu.

## Introduction

Adrenal adenomas are benign tumors that originate from the adrenal cortex and represent the most prevalent type of all adrenal lesions. Adrenal masses occur in approximately 1.4% of the general adult population during abdominal imaging performed for unrelated conditions, with adrenal adenomas accounting for over 75% of these incidental findings [1]. These benign tumors are predominantly non-functioning regardless of age [2], though a notable proportion, approximately 15%, consists of hormonally active lesions, including cortisol-producing adenomas [3]. While overt cortisol secretion leads to *Cushing Syndrome* (CS) with characteristic clinical manifestations, milder forms of cortisol dysregulation, such as *Mild Autonomous Cortisol Secretion* (MACS), have been increasingly recognized. These cases, though lacking overt symptoms, are associated with significant cardiometabolic risks, such as hypertension, diabetes mellitus, and increased mortality [4]. When an adrenal mass is identified, further assessment primarily focuses on two key goals: differentiating between benign and malignant masses, and determining whether the tumor is hormonally active or not-functioning [3,5]. This involves a combination of clinical evaluations and imaging techniques. After providing a brief overview of the general approach to incidentally discovered adrenal masses, this article aims to present a case series and explore the diagnostic strategies and evolving management options for adrenal adenomas, with a focus on the growing role of minimally invasive techniques like *Percutaneous Ablation* (PA) as alternatives to the traditional surgical approaches.

## Materials and Methods

### Patients

This case series examines the outcomes of computed tomography-guided microwave ablation (MWA) performed on three patients diagnosed with functioning adrenal adenomas between July 2021 and June 2024. Written informed consent was obtained from all participants prior to inclusion.

The patients were referred from the outpatient endocrinology clinic at Vilnius University Hospital Santaros Klinikos with confirmed diagnoses of functioning adrenal adenoma. These diagnoses were based on a combination of clinical history, physical examination, specific laboratory tests, and computed tomography (CT) imaging. All patients displayed clinical features consistent with cortisol hypersecretion, which was confirmed by post-suppression cortisol levels.

**Fig. 1:**
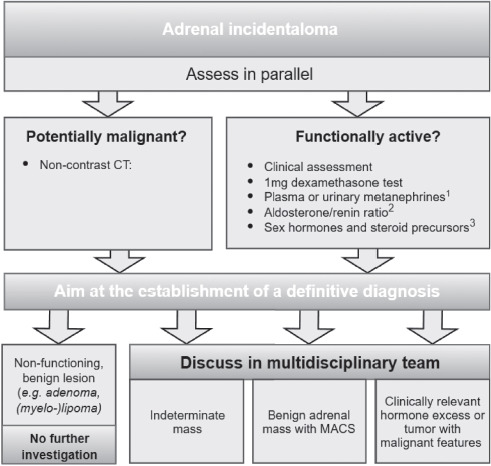
Flow-chart on the management of patients with adrenal incidentalomas, adapted from European Society of Endocrinology 2023 guidelines. ^1^Only necessary in adrenal tumors with Hounsfield unit (HU) > 10 in unenhanced CT. ^2^Only in patients with concomitant hypertension and/or hypokalemia. ^3^Only in patients with clinical, biochemical, or imaging features suggestive of adrenocortical carcinoma [6].

Three patients with functioning cortisol-secreting adrenal adenomas were included in a retrospective study. The inclusion criteria encompassed patients aged 18 or older with a confirmed functioning cortisol-secreting adrenal adenoma, as determined by clinical assessment, specialized biochemical testing, and upper abdominal CT imaging according to *European Society of Endocrinology* clinical practice guidelines on the management of adrenal incidentalomas (Fig. 1) [6]. Lesions were characterized as benign based on CT protocols, with typical nodules showing density of <10 Hounsfield units (HU) on non-contrast CT [6]. Histological confirmation was unnecessary as all cases met clinical diagnostic criteria and demonstrated characteristic CT findings. None of the patients had any primary malignancies.

Due to age, obesity, significant comorbidities and elevated cardiovascular risk (Table 1), none of the patients were considered suitable for radical adrenalectomy, thus making adrenal ablation the preferred alternative. To mitigate the risk of hypertensive crises due to catecholamine release, all patients underwent premedication with an alpha-adrenergic blocker for two weeks prior to ablation [7]. Adrenal venous sampling was conducted in one patient with bilateral adenomas to determine the laterality of excess hormonal secretion and select the target lesion for ablation.

Significant details from patient histories, clinical presentations, laboratory findings, and imaging results are summarized in Table 1.

**Table 1:** Patient characteristics, diagnostic work-up, treatment specifications and post-treatment outcomes

Patient/ Age	Procedure Date	Patient History	Physical Examination	Laboratory Tests	Imaging Tests	Diagnosis	Treatment Procedure	Post-treatment Complications	Follow-up
Case 1 79	July, 2021	PAH, obesity, coronary artery disease, heart failure, osteoporosis	BP: 130/80 mmHg Pulse rate: 69 BPM BMI: 30.4 kg/m^2^ Height: 154 cm Weight: 72 kg WC.: 98 cm	BG: 6.2 mmol/L MCor: 615 nmol/L OnDST: 598 nmol/L (no suppression)	CT: bilateral lipid-poor adrenal adenomas; 6x9 mm lesion on the left side and 16x17mm lesion on the right side	ACTH-independent CS	Microwave ablation at 30 W for 10 min. Procedure duration: 55 min.	Acute adrenal insufficiency (MCor 225 nmol/L)	**At 1 month:** Weight 72 kg, BP 140/80 mmHg, BG 4.75 mmol/L, MCor 179 nmol/L. **At 4 months:** Weight 70 kg, BP 156/76 mmHg, BG N/A, MCor 135 nmol/L. **At 10 months:** Weight 69 kg, BP 186/105 mmHg, BG 5.10 mmol/L, MCor 144 nmol/L. **At 25 months:** Weight 68 kg, BP 150/67 mmHg, MCor 363 nmol/L. **At 32 months:** Weight 65.8 kg, BP 188/80 mmHg, MCor 371 nmol/L.
Case 2 52	September, 2023	PAH, obesity, non-toxic multinodular goiter, insulin resistance, depression	BP: 170/100 mmHg Pulse rate: 86 BPM BMI: 36.8 kg/m^2^ Height: 168 cm Weight: 107 kg WC: 118 cm	BG: 5.66 mmol/L MCor: 317 nmol/L OnDST: 135 nmol/L (no suppression)	CT: 7x11 mm right adrenal adenoma	MACS	Microwave ablation at 30 W in two 3-min cycles. Procedure duration: 100 min.	None	**At 1 month:** Weight 104 kg, BP 140/110 mmHg, BG 6.13 mmol/L, MCor 312 nmol/L. **At 4 months:** Weight 102 kg, BP 117/78 mmHg, BG 6.13 mmol/L, MCor 377 nmol/L, OnDST 47 nmol/L. **At 11 months:** Weight 101 kg. BP 128/80 mmHg, BG 6.23 mmol/L, MCor N/A, OnDST 46 nmol/L.
Case 3 66	June, 2024	PAH, morbid obesity, persistent AF, DMT2, chronic hypokalemia	BP: 147/82 mmHg Pulse rate: 72 BPM BMI: 44 kg/m^2^ Height: 171 cm Weight: 126 kg WC: *N/A*	BG: *N/A* MCor: *N/A* OnDST: 157 nmol/L (no suppression)	CT: 28x19 mm left adrenal adenoma	MACS	Microwave ablation at 40 W for 10 & 5 min, multiple sites. Procedure duration: 120 min.	None	**At 4 months:** Weight 120 kg. BP 140/90 mmHg, BG N/A, MCor 378 nmol/L, OnDST 95 nmol/L.

AH, primary arterial hypertension; AF, atrial fibrillation; BP, blood pressure; WC, waist circumference; BG, blood glucose (serum); MCor, morning cortisol; OnDST, overnight 1 mg dexamethasone suppression test; US, ultrasonography; CT, computed tomography; CS, Cushing’s syndrome; MACS, mild autonomous cortisol secretion; DMT2, Diabetes Mellitus type 2.

### Percutaneous Adrenal Ablation Procedure

**Fig. 2:**
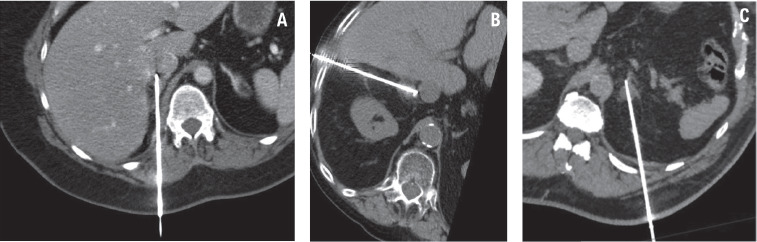
. Intraprocedural reconstructed native or contrast-enhanced computed tomography (CT) images showing the microwave antennas within the adenomas: in the right adrenal gland of the case 1 patient (**A**), in the right adrenal gland of the case 2 patient (**B**) and in the left adrenal gland of the case 3 patient (**C**).

Adrenal ablation procedures were performed under general anesthesia. The patient’s positioning – either on their left side or prone – was determined by the interventional radiologist to optimize access to the adrenal lesion, based on its location and the required angle for a safe and effective approach during MWA. Native or contrast-enhanced control abdominal CT imaging was used to confirm the exact lesion location and plan the needle trajectory. Using ultrasonography (US) and CT guidance, a 14-gauge antenna was inserted through 2 mm skin incisions to target the adrenal adenoma (Fig. 2). MWA was performed at varying power settings and durations, with full details provided in Table 1. Post-procedural CT scans were then performed to assess the immediate success of the treatment and identify any potential complications.

Postoperatively, the patients were transferred to the ward for further monitoring. Treatment efficacy was evaluated through clinical assessments and laboratory tests conducted at various intervals post-procedure.

## Results

The case series included three patients with four adrenal nodules, comprising one male and two females, with a mean age of 65 years (range 52–79). All patients had a history of arterial hypertension and obesity, which compounded their clinical presentation. Two patients were diagnosed with MACS and one with Adrenocorticotropic hormone (ACTH)-independent CS. Tumor sizes ranged from 14 to 28 mm, with a mean of 15.75 mm. All patients underwent a single session of MWA, with two of the three treated lesions located in the right adrenal gland. Immediate post-procedure CT scans showed no enhancement on targeted adrenal adenomas, confirming technical success of all treatments. No procedural complications were encountered.

All procedures were conducted under general anesthesia with orotracheal intubation. Patient positioning on the CT table was determined by the tumor location and laterality. Dorsal access was obtained in two cases with patients placed in the prone position, while transhepatic access was employed in one case with the patient positioned on their left side. The mean total procedure time was 91.7 minutes (range 55–120), and the mean length of hospital stay was 9.3 days (range 1.5–19).

Postoperative outcomes varied among the patients. One of them developed generalized weakness and hypotension, along with a marked decline in morning cortisol levels, leading to a diagnosis of acute adrenal insufficiency. Hydrocortisone therapy was initiated and continued for 28 months until the adrenal function returned to normal. The other two patients had no complications, but small doses of hydrocortisone were prescribed for one patient for 2 weeks to prevent adrenal insufficiency.

Follow-up evaluations demonstrated varying degrees of clinical improvement in all patients, including enhanced well-being and an average weight reduction of 6 kg (range 6–6.2 kg) over a mean follow-up period of approximately 15.7 months (range 4–32 months). However, there were no significant changes in blood pressure management, and all patients continued antihypertensive therapy. Notably, one patient experienced improved blood pressure control with medication. Furthermore, one patient achieved normalization of blood glucose levels.

Pre-procedure cortisol levels varied among the patients, with two showing significant reductions post-treatment. One patient’s morning serum cortisol dropped from 615 nmol/L (22.3 µg/dL) to 135 nmol/L (4.9 µg/dL) within the first four months and remained within the normal range throughout the entire follow-up period of 32 months. Another patient’s post-suppression cortisol level fell from 135 nmol/L (4.9 µg/dL) to 47 nmol/L (1.7 µg/dL) at three months and maintained this level through 11 months of follow-up. The third patient showed only a partial response, with post-suppression cortisol levels decreasing from 157 nmol/L (5.7 µg/dL) to 95 nmol/L (3.4 µg/dL), but these values did not reach the target range, indicating persistent MACS. For this patient, a follow-up 1 mg dexamethasone suppression test is planned to assess cortisol activity over time. In the event of persistently inadequate suppression, repeat CT imaging will be considered to re-evaluate the cause and determine the most appropriate course of further management. For full details, see Table 1.

## Literature Review and Discussion

### Clinical Significance of Adrenal Incidentaloma

Before the widespread use of cross-sectional imaging, adrenal tumors were rare and typically remained undetected until they grew large enough to be detected or led to significant hormonal imbalances. However, with the increasing use of CT and *Magnetic Resonance Imaging* (MRI), adrenal lesions are now frequently discovered incidentally, during scans performed for other purposes [3]. These lesions, named adrenal incidentalomas, have a reported prevalence ranging from 0.35% to 1.9% in CT studies, with autopsy data suggesting a slightly higher rate of 2.3%. Adrenal masses occur with an almost equal frequency in men (45%) and women (55%), with a median age of the diagnosis at around 62 years. These lesions are quite rare in children, accounting for only 1% of all cases in patients under 18 years old [8].

Adrenal tumors are classified into five broad categories: adrenal adenomas and nodular hyperplasia, benign lesions (such as myelolipomas, cysts, and hematomas), adrenocortical carcinomas, malignant tumors (including metastases, sarcomas, and lymphoma), and pheochromocytomas. Among these, adrenal cortical adenomas are the most commonly found incidentalomas, accounting for 83.7% of all benign adrenal tumors, according to a population-based study by Ebbehoj et al. [8]. Adrenal adenomas can range from benign, non-functioning lesions to hormonally active tumors that require therapeutic intervention to prevent complications such as hypertension, metabolic disorders, and cardiovascular risks [3,9]. Once an incidental adrenal mass is discovered, clinicians face two critical questions: 1) *Is the mass benign or malignant?* And 2) *Is it hormonally active or non-functioning?* These factors play a crucial role in determining the management approach, as malignant or functioning mass often require surgical intervention to prevent hormonal imbalances or metastasis [6]. Among functioning adenomas, the most common are those associated with *Primary Hyperaldosteronism* (PH) and CS [10].

### Radiological Assessment of Adrenal Adenoma

CT imaging remains the most widely used and practical modality for assessing the adrenal gland, particularly on the axial plane by sections of f 2.5–3 mm. It characterizes lesions based on intracellular lipids, with glucocorticoid-producing adenomas typically being small, solitary, and well-demarcated, with low attenuation values [11]. Over 70% of adrenal adenomas are lipid-rich, and easily identifiable on imaging [12]. Attenuation values ≤10 HU on an unenhanced CT are reliable markers for lipid-rich adenomas, offering a sensitivity of 71% and a specificity of 98% [13,14]. However, attenuation values >10 HU complicate differentiation from malignant lesions. Contrast-enhanced washout CT (CEW CT) further aids characterization by evaluating perfusion patterns and enhancing lesion visualization, especially with intravenous contrast [1,9,15]. A delayed-phase protocol demonstrates high sensitivity and specificity for adenoma identification. Both lipid-rich and lipid-poor adenomas exhibit similar washout behaviors [1,9,15,16].

While CT is effective, MRI is equally sensitive and can outperform CT in cases with densities between 10–20 HU on unenhanced CT. MRI’s ability to identify intralesional fat by using chemical shift imaging (CSI) is crucial for distinguishing lipid-rich adenomas from malignant lesions, with reliable diagnostic performance [1,9]. Ultrasound (US), while useful for lesion staging and radiation-free monitoring in children and pregnant women, is less sensitive and operator-dependent, with a lower detection rate for masses under 3 cm [1,17].

Recent imaging advances, such as texture analysis from CT and MRI, enhance differentiation between malignant and benign lesions. Multidisciplinary approaches involving nuclear imaging and endocrine testing are recommended when initial assessments are inconclusive, enabling accurate identification of adrenal adenomas.

### The Role of Cortisol Assessment

Adrenal incidentalomas frequently show biochemical evidence of hormone excess, with cortisol overproduction being the most common. Approximately 50–60% of adrenal cortical adenomas exhibit abnormal cortisol secretion, contributing to ACTH-independent CS [18–20]. MACS, a milder form of cortisol excess without overt CS symptoms, is more common in individuals over 50, particularly women, and is associated with metabolic syndrome features such as obesity, diabetes, dyslipidemia, and hypertension [4,6,21–24]. It is more likely in bilateral or larger adrenal tumors but can also occur in smaller ones [25,26].

**Fig. 3:**
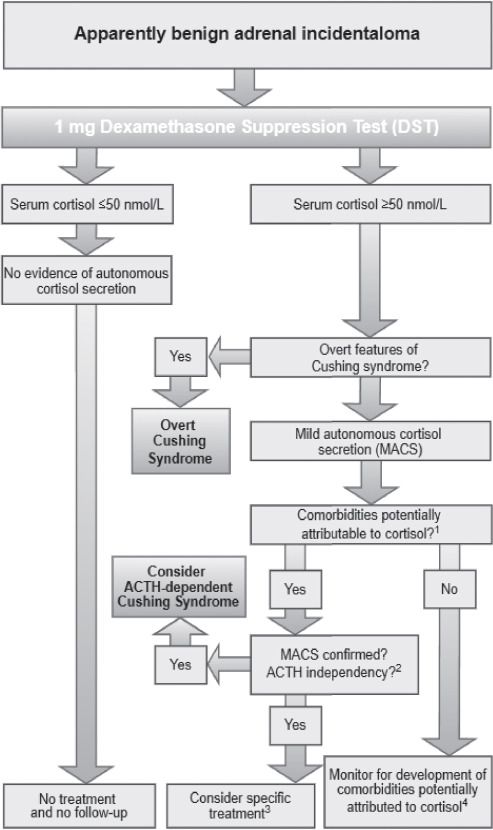
Flow-chart on assessment and management of mild autonomous cortisol secretion (MACS) in patients with adrenal incidentalomas, adapted from European Society of Endocrinology 2023 guidelines. ^1^Comorbidities include diabetes mellitus, hypertension, dyslipidemia, or osteoporosis. ^2^Defined by low/suppressed morning plasma ACTH. ^3^Surgery is considered when comorbidities are progressive, treatment-resistant, cause age-inappropriate end-organ damage, are atypical for age or family history, or when multiple comorbidities are present. ^4^Annual clinical reassessment of comorbidities potentially attributable to cortisol is recommended [6].

The evaluation of adrenal incidentalomas involves clinical assessment and targeted biochemical testing for hormone excess, which was performed in our patients in accordance with the latest clinical practice guidelines by European Society of Endocrinology (Fig. 3). Pheochromocytoma was ruled out with plasma metanephrines, though masses ≤10 HU on unenhanced CT are unlikely to require testing. Patients with hypertension or unexplained hypokalemia had their aldosterone-to-renin ratio measured. Excess cortisol production was assessed via the 1-mg overnight dexamethasone suppression test (1-mg DST), with MACS diagnosed if post-test cortisol remained >50 nmol/L (1.8 μg/dL). Further testing included ACTH measurement and repeat 1-mg DST. In the light of this, it is essential that all patients diagnosed with MACS undergo a thorough re-evaluation in order to identify any potential signs or symptoms of overt Cushing syndrome that may have been missed during the initial assessment [6,26–31].

MACS is recognized as a condition linked to an increased cardiovascular risk, including hypertension, obesity, diabetes and higher mortality [19,22–24]. Treatment decisions should consider age, health status, and comorbidities. While some studies suggest that surgery may improve the outcomes, no large-scale trials have confirmed this yet, making individualized management essential [6,22–24,26,32]. Further research is needed to refine treatment strategies and identify patients who would benefit most from surgery.

### Minimally Invasive Alternatives to Adrenalectomy: The Role of Percutaneous Ablation

Over the past decade, minimally invasive technologies like PA have emerged as promising and effective alternatives for patients unable or unwilling to undergo surgery. These techniques offer several advantages, including shorter procedure durations, reduced blood loss, less postoperative pain, and shorter recovery times compared to traditional surgical approaches [33–35]. PA is particularly beneficial for high-risk patients, such as those with coexisting conditions or previous surgeries, who may not be candidates for more invasive procedures. Furthermore, PA has proven effective in treating functional adrenal tumors, such as pheochromocytomas and aldosterone-producing adenomas, making it a viable alternative to adrenalectomy for managing endocrinopathy (36–39). Given these advantages, various ablation techniques, including hyperthermic methods such as *Radiofrequency Ablation* (RFA) and MWA, alongside the hypothermic approach of cryoablation, have been developed and refined to optimize the treatment of adrenal tumors [37]. MWA and RFA utilize heat to induce coagulative necrosis in adrenal tumors, with MWA employing electromagnetic microwaves and RFA using alternating electrical currents. Both techniques provide advantages over the traditional surgery, including reduced pain, minimal scarring, and quicker recovery times. However, precise targeting is crucial to avoid damage to adjacent tissues [40–42]. Cryoablation, in contrast, destroys tumor cells by freezing them, allowing for clear visualization of the ablation zone under imaging guidance. This method is particularly beneficial for monitoring the extent of ablation and avoiding injury to nearby structures. Each technique has its unique benefits and considerations, and the choice of method should be tailored to the patient’s clinical scenario [43]. A key concern is the potential for harm to nearby organs located in close proximity to the adrenal glands. Recent studies have incorporated the use of hydrodissection (saline infusion) alongside MWA to separate non-target tissues and organs from the ablation area, thus creating a protective barrier between the tumor and adjacent structures [44,45]. Positive outcomes require at least 10 mm of separation during the procedure, but the use of an additional catheter for saline infusion increases the invasiveness of the treatment.

Percutaneous ablation (PA) of adrenal tumors is a generally safe and effective procedure, though it carries certain risks and complications. One significant perioperative concern for hormonally active adenomas is the risk of hypertensive crises resulting from the sudden and massive release of catecholamines. This phenomenon is typically observed shortly after the initiation of hyperthermal ablation (RFA and MWA) or during the thawing cycle in cryoablation, with previously reported incidences ranging from 0% to 62.5%. Premedication with alpha-adrenergic blockers is typically administered, and it was administered to our patients as well, 10–14 days before surgery to mitigate the risk, thereby successfully avoiding this life-threatening complication [46]. According to the guidelines of the European Society of Endocrinology, perioperative glucocorticoid treatment at surgical stress doses is recommended for all patients undergoing surgery with post-suppression cortisol >50 nmol/L (1.8 µg/dL), because MACS and ACTH-independent CS could lead to adrenal insufficiency after the removal of the adrenal source of cortisol. Postoperative glucocorticoid replacement (preferably with hydrocortisone) is encouraged when no suppression is documented in preoperative post-suppression cortisol levels, and it should continue until the recovery of hypothalamic-pituitary-adrenal (HPA) axis function has been confirmed [6]. Two of our patients received hydrocortisone replacement therapy for varying durations (2 weeks and 28 months), depending on their individual recovery and HPA axis function assessment. Technical complications, such as pneumothorax, hemorrhage, vascular thrombosis, and visceral perforation, can occur because of the adrenal gland’s anatomical location and proximity to critical structures. Pneumothorax usually develops in ablation needle which is inserted when implementing the trans-pulmonary approach, accounting for up to 25% cases in previously reported studies [46].

Recent studies have explored the efficacy of PA as an alternative to surgical adrenalectomy for treating benign adrenal masses. A systematic review and meta-analysis by Skribek et al. (2024) reaffirmed that while *Laparoscopic Adrenalectomy* (LA) remains the gold standard treatment for aldosterone-producing adenomas, PA is a viable option for patients who are not surgical candidates, including those with extreme obesity, prior abdominal surgeries, coagulopathies, or cardiopulmonary diseases. However, PA has been associated with a higher risk of hypertensive crises and a lower biochemical success rate in resolving hypertension compared to LA [33]. Nevertheless, several recently published studies reported no significant differences in other major and minor complication rates between the two procedures [33–35]. Blood pressure management outcomes, however, remain inconsistent. While Skribek et al. reported inferior blood pressure control with PA [33], Chen et al. (2021) observed greater reductions in both systolic and diastolic blood pressure following PA compared to LA [34]. Meanwhile, Ma et al. (2024) found no significant difference in systolic blood pressure outcomes but reported superior diastolic blood pressure control with LA [35]. There is limited documentation on adrenal ablation use specifically for cortisol-secreting adrenal adenomas and the treatment of MACS and CS. Nevertheless, several studies have reported positive outcomes, showing notable improvements in both clinical and laboratory findings. For instance, a study from 2007 demonstrated that the majority of patients treated with adrenal ablation (specifically RFA) showed normalization of cortisol and ACTH levels, accompanied by symptom relief such as an improved body weight and hypertension, and tumor shrinkage or complete absence during follow-up [47]. However, both a study and a case report published in 2011 and 2012, respectively, observed a temporary adrenal insufficiency which developed in some patients following the ablation of cortisol-secreting adenoma, which was effectively managed with hydrocortisone therapy. Notably, tumor recurrence or any signs of hypercortisolism were not observed after treatment [48,49]. Another study published in 2013 reported a high success rate (91%) in patients with CS, with the exception of two patients who experienced mild periprocedural complications such as bradycardia and minor pneumothorax, which resolved quickly [50]. Additionally, more recent studies have shown that RFA leads to normalization of cortisol and ACTH levels in many cases, with some patients maintaining long-term improvements without adverse effects [51,52]. Variations in ablation outcomes across different studies may be attributed to factors such as the specific medical center and the level of experience of the interventional radiologist conducting the procedure. Overall, the majority of studies comparing PA and surgical adrenalectomy conclude that adrenalectomy remains the optimal treatment approach for adrenal tumors, while PA serves as an effective alternative treatment option for patients ineligible for surgery.

The purpose of this report was to document the efficacy of percutaneous ablation as an alternative treatment for a small group of patients for whom surgical therapy was not feasible due to either morbid obesity or a lack of consent. In all of the cases reported on here, adrenal ablation made some improvements regarding clinical symptoms such as the body weight, diabetes, hypertension, overall well-being, and laboratory findings, such as blood glucose, morning cortisol and post-suppression cortisol levels. The findings of our case report series align with previous research, supporting the notion that adrenal ablation can serve as an effective treatment for hypercortisolism when other methods have proven unsuccessful or are not feasible.

## Conclusions

Ultimately, PA presents a promising alternative treatment for adrenal adenomas in patients who are not candidates for surgery due to comorbidities or personal preferences. It offers advantages such as shorter procedure durations, reduced blood loss, and quicker recovery. While PA has shown efficacy in managing functional tumors and endocrinopathies, its success in blood pressure control remains inconsistent, and concerns persist regarding hypertensive crises. Further high-quality studies are needed to refine patient selection and establish the long-term role of percutaneous ablation in clinical practice.
